# Perceptions and Preferences of Patients with Terminal Lung Cancer and Family Caregivers about DNR

**DOI:** 10.7759/cureus.271

**Published:** 2015-05-27

**Authors:** Naseer Ahmed, Michelle Lobchuk, William M Hunter, Pam Johnston, Zoann Nugent, Ankur Sharma, Shahida Ahmed, Jeff Sisler

**Affiliations:** 1 Department of Radiation Oncology, CancerCare Manitoba, Cancer Care Manitoba, University of Manitoba, Canada; 2 Faculty of Nursing, Cancer Care Manitoba, University of Manitoba, Canada; 3 Radiation Oncology, Tom Baker Cancer Centre; 4 Department of Nursing, Cancer Care Manitoba, University of Manitoba, Canada; 5 Department of Epidemiology and Cancer Registry, Cancer Care Manitoba, University of Manitoba, Canada; 6 Cancer Care Manitoba, Cancer Care Manitoba, University of Manitoba, Canada; 7 Family Medicine, Cancer Care Manitoba, University of Manitoba, Canada

**Keywords:** dnr, end of life care, patients and care givers, terminal lung cancer

## Abstract

Background: Patients with terminal lung cancer and their families are challenged and stressed with the end of life discussions. Do Not Resuscitate (DNR) orders are a critical part of such discussions.

Objective: To understand the perceptions and preferences of patients with terminal lung cancer and their family caregivers around DNR discussions. .

Methods: Our quantitative component consisted of a pen-and-paper questionnaire that was followed by a ‘think aloud’ process to capture perceptions of participants in response to questionnaire items. Qualitative methods included content analysis and constant comparison techniques to identify, code, and categorize primary themes arising from ‘think aloud’ responses.

Results: In this pilot study, 10 patients with advanced stage lung cancer and nine family caregivers were enrolled from one tertiary cancer care centre. Three major themes and several sub-themes were identified reflecting participants’ psychosocial environment, emotional responses to DNR discussions, and suggestions to improve DNR discussions. Most of the time, both patients and caregivers perceived a supportive environment within their family unit. Some patients were uncertain about their disease extent but most had entertained thoughts about prognosis and DNR status prior to having a discussion with their physician. A range of situations stimulated the DNR discussion. Most patients were uncertain about identifying the most appropriate health care provider (HCP) for DNR discussion. While participants found DNR discussions distressing, patients maintained hope in the face of accepting a terminal diagnosis. There were mixed feelings about the reversibility of a DNR decision and concerns about the care of the patients after being stated as DNR. Participants desired their HCP to be emotionally sensitive, knowledgeable, respectful, and straightforward.

Conclusions: Most participants were open about their experiences with psychosocial supports and emotional reactions and made suggestions to HCP to improve DNR discussions. Further examination in larger longitudinal studies is required to validate the observations in the current study.

## Introduction

Patients diagnosed with terminal lung cancer have a rapidly declining performance status and are frail and emotionally vulnerable. Both patients and their families are confronted with the difficult end of life (EOL) discussions, and decisions sometimes need to be made within days after receiving a terminal diagnosis. Do Not Resuscitate (DNR) orders are a significant part of EOL discussions [1]. We recently published a report describing perceptions and preferences of patients, their family caregivers (CGs), and their health care providers (HCP), with respect to their understanding of and the appropriate time to discuss DNR [2]. In the current article, the investigators report study findings from their cross-sectional exploration of cognitive and emotional perceptions and preferences of patients and their CGs when engaged in DNR discussions.

## Materials and methods

In this pilot study, 10 patients and nine CGs who met the eligibility criteria were recruited from Cancer Care Manitoba (CCMB) in Canada where tertiary and multidisciplinary care are provided to approximately 800 newly diagnosed lung cancer patients per year. Once considered incurable, patients are given the option to enroll into a palliative care program. Patients must have a DNR status to be eligible for this program. Patients with a pathological diagnosis of either non-small cell or small cell lung cancer and incurable disease, who did not exhibit confusion, and who were not to receive further curative therapy, were eligible. Additional eligibility criteria included a discussion with the HCP around DNR, and having a CG who was willing to participate in the study. A CG was identified by the patient as the individual who assisted most with his or her care and was most involved with the patient in the DNR status decision-making was eligible to participate in the study.

Quantitative and qualitative methods were used to capture perceptions of study participants. A dedicated research nurse was trained on the interview technique before she conducted interviews of the participants.

A written informed consent was obtained from the participants. Prior to commencing the study’s protocols, the investigators obtained ethics approval from the Research Ethics Board University of Manitoba (approval #H2011: 227).

Quantitative Component

Study participants completed respective pen-and-paper versions of an investigator-developed questionnaire for patients and CGs. In these tools, we collected socio-demographic and medical information, which were verified with the patient’s medical record with permission from the patient in the written informed consent. 

Qualitative Component

We employed a ‘think aloud’ process where participants were prompted to immediately verbalize their thoughts when responding to items in the investigator-developed questionnaire on DNR status and DNR discussions [2]. In response to our prompts, we were able to glean an understanding of their level of understanding, personal wishes, triggers, timing, appropriate setting, emotional experiences, appropriate health care provider, and extent of family dialogue on DNR decisions. Interviews were audio-recorded and transcribed for interpretation and analyses of the key themes. A more detailed description of the interview process is captured in another article [2].

Data Analyses

Demographic and medical characteristics, as well as subgroup responses to questionnaire items, were captured by descriptive statistics (Tables 1-2).

**Table 1:** Patients (n = 10) Characteristics

Characteristics	Patients (n)
Median age	67.5 years
Gender	
M	6
F	4
Religious affiliation	
Affiliated to some religion	9
Not affiliated to any religion	1
Histological diagnosis of lung cancer	
Non-small cell	9
Small cell	1
DNR status	
DNR	9
No DNR	1
Brain metastasis	
Present	3
Absent	7
Whole brain radiation prior to interview	
Received	2
Not received	8
Location of the interview	
Outpatient clinic	8
In hospital	2
CGs accompanying the patient during study interview	
Yes	8
No	2

**Table 2:** Caregivers (CGs) (n = 9) Characteristics

Characteristics	CGs (n)
Median age	67.5 years
Gender	
M	3
F	6
Relationship	
Spouse	5
Parents	1
Sibling	2
Friend	1
Religion	
Anglican	2
Lutheran	1
Greek Catholic	1
United Church	4
None	1
Culture/ethnicity	
Scottish, Italian	1
French, Quebec	1
Icelandic Irish	1
Irish English	1
Scottish, German	2
Ukrainian	2
Ukrainian polish dutch	1
Education	
High school or less	6
University	3
Income	
40-80,000K	3
<20K	2
No answer	2
Occupational status	
Working part-time or full-time	3
Retired	6
Frequency of contact with patient	
Living with patient	7
Visiting weekly	2

Qualitative Component

The trained medical transcriptionist transcribed audiotapes of participants’ ‘think aloud’ responses to questionnaire items and semi-structured interview questions. Content analysis and constant comparison techniques were employed by the investigators to identify, code, and categorize primary themes in the transcribed data [3].

Themes and Coding

Themes and sub-themes from transcribed data obtained from both groups of participants were extracted by three members of the investigative team (NA, WH, and ML). To address qualitative rigor, a detailed audit trail was kept to ensure accurate research procedures were followed. Each investigative team member who independently reviewed the transcribed data kept memos to record theoretical and interpretive insights. The investigative team discussed prominent themes and engaged in a constant comparison of emergent categories. The investigative team met until we achieved consensus on prominent themes and sub-themes. Themes and sub-themes were further refined and reflected in a coding template.

## Results

### Participant characteristics

Study participants consisted of 10 patients with advanced stage lung cancer and nine CGs from the lung cancer clinic. One CG could not be interviewed due to logistic reasons and was excluded from analysis. The majority of patients were: male, a median age of 67.5 years of age, had some religious affiliation, a pathologically confirmed diagnosis of non-small cell lung cancer, an identified DNR status, no brain metastases, and had not received whole brain radiation prior to being interviewed (Table 1). Most caregivers were: female, retired, a median of 67.5 years of age, the spouse who lived with the patient, affiliated with the United Church, varied in ethnic background, had reported having less than or high school education, and earned either between $40,000 to 80,000, or less than $20,000 in annual income (Table 2). Most patients and their CGs were interviewed in the outpatient clinic (Table 1).

Three major themes, along with several sub-themes, were identified as central to DNR discussions: patients’ and CGs’ psychosocial environment, patients’ and CGs’ emotional responses while engaged in DNR discussions, and suggestions to improve DNR discussions.

### Psychosocial environment

The first main theme of *‘Psychosocial environment’ *portrays reflections and insights of patients and CGs about their mutual interactions in the context of impending death. Five sub-themes describe patients’ and CGs’ perceptions about their relationships and supports, self-awareness, a previously thought through DNR decision, triggers of the DNR discussion, and the involvement of others in DNR discussions.

Sub-theme: Relationships and Supports

This sub-theme reflected a sense of support perceived by patients as coming from mainly spousal caregivers, siblings, and/or friends of the patients who lived with them. Most of the time, both patients and CGs reported having a supportive environment within their family unit (Table 3). When presenting for the scheduled interview, it was noted that most patients were accompanied by their CGs (Table 1).

**Table 3:** Psychosocial support perceived by the patients (n = 10) and CGs (n = 9)

	CGs	Patients
Always	6	6
Frequently	2	2
Sometimes	1	1
Rarely	0	1

Sub-theme: Self-awareness

This sub-theme reflected the degree of understanding of the extent of the disease and prognosis by most participants. A small number of patients expressed uncertainty about the terminal nature of their disease (Table 4). In spite of being told by the physician about their disease stage, some patients seemed to be uncertain about their poor prognosis. As one patient stated *“I don’t know where I’m at? I mean they told me I have Stage IV”. *Although some patients appeared* uncertain*, at the same time they also seemed aware that their disease is terminal.

**Table 4:** Patients awareness about the extent of their disease and prognosis

Perception	Patients (n)
“Don’t know/incurable"	1
“Incurable”	2
“Stage 4”	1
“Not early stage, spread to bone, it's aggressive, it could take off like any time.”	1
“Not sure - at first they thought it was stage 4, then they thought stage 3. Just about the end of the line there.”	1
“One Dr. told me that I have terminal cancer, 2-3 months, but that Dr. wasn't sure. My oncologist told me 6 months.”	1
“Don't know.”	1
“Don't know, but I knew it was bad.”	1
“Not sure.”	1

Sub-theme: A Previously Thought Through DNR Decision

In this sub-theme, patients and/or family members have entertained thoughts about prognosis and DNR status before a formal DNR discussion with their physician. Nine of 10 patients had a predetermined DNR status and seven of 10 patients had previously considered DNR prior to discussing the topic with a health care provider. One caregiver stated: *“It’s something I guess you [patient] thought … I thought about it. …” *that was followed by the patient’s response, “*Yeah, I don’t want to die at home…"*. That appeared to be an important if not influential aspect in this individual’s decision-making about his or her DNR status. Although many patients and CGs had engaged in thought processes about DNR prior to meeting with their HCP, only five of 10 patients had previously signed a document related to their DNR status.

Sub-theme: Triggers of DNR Discussion

This sub-theme depicts triggers that prompted DNR discussions with the HCP and between patients and their CGs. Table 5 captures a range of situations that stimulated DNR discussions, including admission to hospital, a family member who wanted to talk about DNR, the HCP initiated the conversation, the results of pathology or scans, and brain metastasis. When prompted for further triggers that initiated DNR discussions, most patients described a “situation of emergent care”. One patient stated*: “When I came in the first time, they talked about it (DNR) before they started any treatment”*. The accompanying family member added to clarify: “*Which was after having been diagnosed…you didn’t know what the stage of cancer was at that time, but you were experiencing speech problems and had an emergency CT scan and they found complications with the brain*” (brain metastasis).

**Table 5:** Triggers to initiate DNR discussion

Trigger	Patients (n)
Admission to the community hospital	1
Brother brought it up	1
Radiation oncologist initiated the discussion	1
Pathology results	1
Patient himself initiated the discussion	2
Results of CT scan at a scheduled visit	1
Diagnosis of brain metastasis	2
Family physician clarified how patient wanted to be cared for	1

Sub-theme: Who Should be Involved in the DNR Discussion?

Patients and CGs indicated their preference for the HCP and/or family member to be involved in DNR discussions. Most patients had their discussions about DNR with their physician. When specifically asked who else should be present during these discussions, most patients and their CGs preferred to have a family member or a support person present in addition to a physician. However, when patients and CGs were interviewed to describe their preference for the most appropriate HCP to initiate DNR discussions, the following categories of responses were identified: uncertainty and depends on the patient’s situation.

The category of “uncertainty” captures statements by patients indicating their uncertainty about the appropriate HCP to discuss DNR. As one patient stated, “*You know I probably didn’t know who to put … I don’t know who would … Dr. X (oncologist)? He’s not my family physician.”  *The second category, “depends on patient situation” reflects family members’ reliance on the ‘context’ of the patient’s condition to decide who is appropriate and ‘when’ to have the DNR conversation with the patient. As one CG stated, “*Well, it would be the medical resident or a physician who felt that the condition warranted the discussion. You know, so it was trust in the medical system per se and I would presume in that emergency situation that discussion was with a resident, but I may be incorrect about that.”*

### Emotional response to DNR discussions

The second main theme of ‘*Emotional response to DNR discussions’* captures a qualitative account about the degree of distress that patients and CGs perceived they experienced (ranging from ‘a little’ to a ‘moderate’ amount on the investigator-developed emotional response survey item) when engaged in DNR discussions (Table 6). Most patients perceived that a DNR status would not compromise their care and felt that a DNR decision could be reversed. The following sub-themes reflect patients’ emotional and cognitive acceptance of the situation.

**Table 6:** Emotional distress perceived by DNR discussions

	Patients (n)	Caregivers (n)
A little	4	7
Moderate amount	3	1
Not at all	3	-

Sub-theme: Acceptance of the Situation and Prognosis

The first sub-theme reflects patients’ “Acceptance of the Situation and Prognosis” where one patient stated, “*I just feel I’ve got it, what can I do*?” A “fighting spirit” reflected an attitude adopted by patients to deal with the disease and prognosis. One patient stated, “*So I’m going to fight it. So there was no use being upset at that point; just crash on.”  *

Sub-theme:  Definitely Distressing in an Emergent Situation

The sub-theme of “Definitely Distressing in an Emergent Situation” reflects patients' and CGs' upset and anxiety when DNR is discussed in an emergency or unanticipated situation. One CG stated, “*Yeah, I think that it’s obviously distressing, the whole event that led to it was a distressing event in terms of entering Emergency with a prognosis that there were serious risks.”  *

Sub-theme:  Mixed Feelings

The sub-theme of “Mixed Feelings” reveals a mixture of hope tempered by an admission of imminent death by study participants. One patient stated, “*I guess I’m sort of mixed feelings… I said we’re going to beat it. So I’m still in that mind frame and I don’t really know if I want to know whether I have a month or six months or a year.”*

Sub-theme: Nobody Wants to Talk About It 

The sub-theme of “Nobody Wants to Talk About It” reflects statements by study participants indicating the taboo nature of talking about DNR. One patient shared, “*I guess it’s probably something nobody wants to talk about, but so you know I don’t know how the reaction of how some people would react to it.” *

Sub-theme: Uncertain About His/Her Right to Change One's Mind About DNR Status

The sub-theme of “Uncertain About His/Her Right to Change One’s Mind About DNR Status” across the illness trajectory reflects the participants’ lack of understanding about being able to reverse one’s decision about DNR status as time goes on. One patient explained, “*Well, I don’t know? If I am down the road and I’m that sick, I don’t think I want to be revived *…”

Sub-theme: Expectations of Same Care

The last sub-theme of “Expectations of Same Care” reflects the participants’ beliefs that regardless of the DNR status, the patient will continue to receive quality care. One patient shared, *“I would like to get the very best of the same care that I’ve had, only in a different manner, you know. It would be a different … I’ve had wonderful care.”*

### Suggestions to improve DNR discussions

The third main theme, ‘*Suggestions to improve DNR discussions’*, reflects patient and CG recommendations on how to improve their comfort while discussing DNR and are reflected in five sub-themes.

Sub-theme: Pay Attention to Delivery

The first sub-theme, “Pay Attention to Delivery”, speaks to the participants felt the need for a sensitive approach and assurance of attentive support during the DNR discussion.  One patient stated,* “I have found on occasion one of the doctors to be very blunt. I think there’s different ways that you can come at it and I felt that this way has been not like Dr. X’s”*. A caregiver added*, “I don’t know. I just think that make sure that those people are supportive to the individual if they’re there are there and present.”* 

Sub-theme: Respect and Awareness of Patient’s Wishes

Sub-theme two, “Respect and Awareness of Patient’s Wishes”, reflects the wishes of participants for health care providers to engage in a patient-centered approach by paying attention to the belief, values, and desires of patients regarding DNR. One caregiver stated, *“He [oncologist] understand what X [patient] would like to accomplish.”* 

Sub-theme: Be Straightforward, Honest but Polite

The third sub-theme, “Be Straightforward, Honest but Polite”, describes for honest and timely communication that is respectful toward the patient’s need for clear-cut information. Different patients stated*:  “Be frank and blunt…Just be straight to people”, “Start talking to people they are health, before they get sick”, and “There’s no way to whitewash it or do anything else but offer reassurance support.”* 

Sub-theme: Expect the Physician to be Knowledgeable and Sensitive​

The fourth sub-theme, “Expect the Physician to be Knowledgeable and Sensitive”, explains participants’ desire for sound medical knowledge that is shared in a caring manner while considering the patient’s unique situation. One patient stated, *“Be informed what kind of condition the patient is in.”*

Sub-theme: Understand the Physician's Viewpoint

The final sub-theme, “Understanding the Physician’s Viewpoint”, reflects the participants’ desire for physicians to be transparent about their understanding of DNR. One caregiver explained, *“I suppose one of the things that might have been discussed to seek clarification would be the physician’s interpretation of what DNR means … You know there is a grey area about just the scope of what DNR means.” *

## Discussion

Optimizing the quality of life (QOL) of patients with incurable lung cancer requires attention to both physical symptoms (e.g., pain) and emotional distress regardless of the DNR status [4]. Our study is a reflection of how terminally ill lung cancer patients and their CGs feel and react when confronted with DNR discussions. In a meta-analysis, including 43 studies from 14 countries with terminal cancer patients, an enhanced sense of psycho-spiritual well-being was the key factor influencing the psychosocial well-being of the patients. Being aware of the prognosis and presence of family support contributed positively while emotional distress and hopelessness had an adverse effect on the emotional health of the patients [5].

Patients and CGs in our study desired a supportive health care environment and they preferred to have a family member present during the discussion. Emotional and physical supports from family members have profound effects on the QOL of dying patients [6]. The National Consensus Project for quality palliative care requires documentation of relationships and existing social networks as part of the social assessment by HCP for palliative care [7].

In our study, although some patient participants expressed uncertainty about the extent of their disease, they accepted the terminal nature of their cancer. It is difficult to explain this somewhat contradictory finding. However, terminally ill patients may differ from each other in the extent that they understand their disease extent and their prognosis [5, 8]. In a Canadian study with 200 advanced cancer patients, those who did not acknowledge their prognosis were three times more likely to be depressed than those who had some acknowledgment of their prognosis [9]. Most patients desire to know their prognosis [1], which supports our study findings of participants’ desire for honest and direct communication about their terminal state.

Most patients in our study held preconceived thoughts about their resuscitative status while other patients described acute situations that triggered DNR discussions. In a study with 1,081 colorectal and lung cancer patients, 55% EOL care discussions occurred during an emergency room visit or a hospital admission [10].

Most of the current study’s patients had DNR discussions with their oncologist or a family physician and usually in the presence of a family member. On the other hand, many patient participants expressed uncertainty when they stated, “depending on the situation” as to who they would identify as the appropriate HCP to initiate DNR discussions. In a Canadian study with 429 patients who visited their physician, 86% identified the family physician as their preferred HCP to discuss DNR, regardless of their DNR status [11]. Considering the uncertainty of our patients about their preferred HCP for DNR discussions, perhaps more collaboration is required between oncologists and the family physicians.

Both patients and CGs found DNR discussions somewhat distressing while maintaining hope and accepting the reality of being terminal. There were mixed feelings about the reversibility of a DNR decision and concerns about the care of patients after the DNR status had been decided. It is plausible that patients may acknowledge the terminal nature of the disease (cognitive acceptance) with or without accepting it at the emotional level. Cognitive and emotional acceptance of having an incurable and terminal cancer can have both a unique and a synergistic effect on EOL outcomes and are determined in part by how the prognosis was communicated by the HCP and how the patient perceives this communication [1]. Patients with only cognitive acceptance may be less peaceful and more symptomatic. On the contrary, emotional acceptance of the terminal nature of the disease is associated with feeling less terrified and more supported by others [12-13]. Furthermore, emotional ‘acceptance’ of the terminal nature of the disease is not a static event with the possibility that the patient may change his or her decision about the DNR status over time, which requires sensitivity understanding on the part of the health care team.

Both patients and their CGs desired their HCP to be emotionally sensitive, knowledgeable, and straightforward in his/her communication with a clear interpretation of DNR. This observation is in concordance with literature where patients described their desire for complete information, to be treated as a unique individual, and for trust in the competence of their physicians [14-18]. 

Limitations

Our pilot study sample size was too small for rigorous statistical analyses of quantitative responses. Themes and sub-themes reflected in this study are limited to the perceptions and preferences of patients and CGs who attended a single mid-prairie Province cancer care clinic located in an urban setting. 

## Conclusions

This cross-sectional pilot study uniquely presents in-depth analyses of thoughts and feelings held by patients with terminal lung cancer and their CGs in a tertiary academic center when engaged in DNR discussions. Observations and themes depicted in this study require further exploration in a larger and more comprehensive study. Regardless, we found that most patients and CGs were willing to talk openly about their experiences with psychosocial supports and emotional reactions that led to their recommendations for HCPs to improve DNR discussions in ways that support their unique desires, preferences, and values when dealing with terminal illness.
